# Accelerating the Worldwide Adoption of Sugar-Sweetened Beverage Taxes: Strengthening Commitment and Capacity: Comment on "The Untapped Power of Soda Taxes: Incentivizing Consumers, Generating Revenue, and Altering Corporate Behavior"

**DOI:** 10.15171/ijhpm.2017.127

**Published:** 2017-10-29

**Authors:** Phillip Baker, Alexandra Jones, Anne Marie Thow

**Affiliations:** ^1^Institute for Physical Activity and Nutrition, School of Exercise and Nutrition Sciences, Deakin University, Geelong, VIC, Australia.; ^2^The George Institute for Global Health, University of New South Wales, Sydney, NSW, Australia.; ^3^The Charles Perkins Centre, University of Sydney, Sydney, NSW, Australia.; ^4^Menzies Centre for Health Policy, Charles Perkins Centre, University of Sydney, Sydney, NSW, Australia.

**Keywords:** Sugar-Sweetened Beverages, Taxes, Political Priority, Capacity, Framing

## Abstract

In their recent article Roache and Gostin outline why governments and public health advocates should embrace soda taxes. The evidence is strong and continues to grow: such taxes can change consumer behavior, generate significant revenue and incentivize product reformulation. In essence, such taxes are an important and now well-established instrument of fiscal and public health policy. In this commentary we expand on their arguments by considering how the worldwide adoption of such taxes might be further accelerated. First, we identify where in the world taxes have been implemented to date and where the untapped potential remains greatest. Second, drawing upon recent case study research on country experiences we describe several conditions under which governments may be more likely to make taxation a political priority in the future. Third, we consider how to help strengthen the technical and legal capacities of governments to design and effectively administer taxes, with emphasis on low- and middle-income countries. We expect the findings to be most useful to public health advocates and policy-makers seeking to promote healthier diets and good nutrition.

## Introduction

Roache and Gostin’s article in this journal presents a compelling rationale for *why* governments and public health advocates should embrace soda taxes.^1^ A strong and growing body of evidence demonstrates that such taxes can change consumer behavior, generate significant revenue for cash-strapped governments, and incentivize product reformulation by manufacturers. The recent addition of sugar-sweetened beverage (SSB) taxes to the menu of policies recommended in the World Health Organization’s (WHO’s) Global Action Plan for the Prevention and Control of Non-Communicable Diseases 2013-2020, further recognizes this evidence and adds to a growing global mandate for action.^2^

In this commentary we expand on Roache and Gostin’s arguments by considering *how* the worldwide adoption of such taxes might be further accelerated through strategic action by public health actors. The term ‘soda’ may be easier to explain to general audiences,^3^ but for the purpose of this paper we adopt SSBs to acknowledge the broader range of products taxed in many jurisdictions (eg, juices, milk-based beverages and non-carbonated drinks) and because tax policies with a public health purpose should ideally apply to all possible beverage substitutes.^4^

## Where Is the Untapped Public Health Potential Greatest?

**Figure 1:**
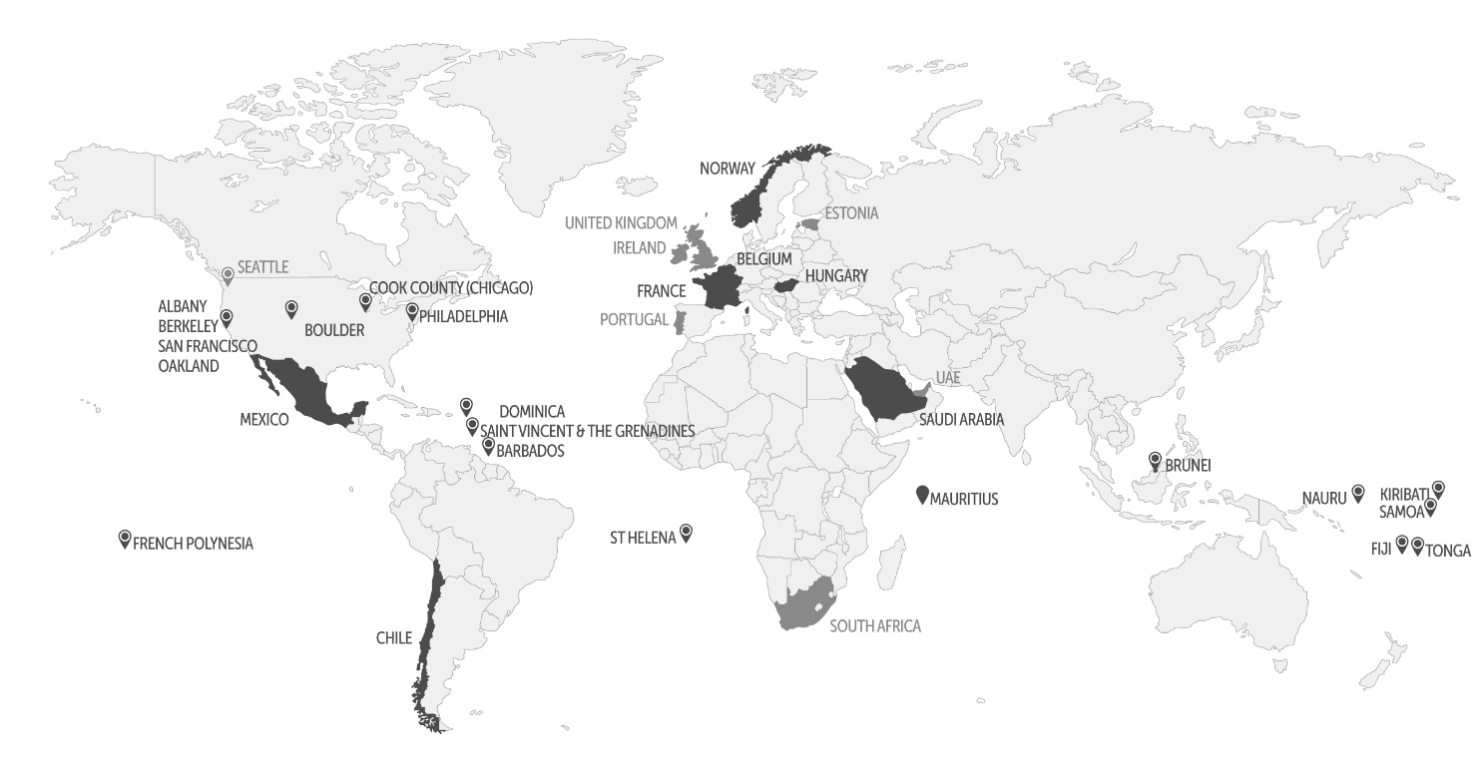
Jurisdictions With Existing (Dark Grey) and Forthcoming (Light Grey) SSB Taxes.
Note: This figure was prepared using data from the NOURISHING database,^9^ academic references and market reports. Given the rapid development of policies and involvement of multiple levels of government, it may not include all jurisdictions with health-related SSB taxes.

Abbreviation: SSB, sugar-sweetened beverage.

Roache and Gostin provide selected country examples where public-health taxes on SSBs have already been enacted. Encouragingly, the full list is in fact far greater and growing. It now includes 19 countries (with six soon to follow), and nine US municipalities (Figure 1). Most of these countries have higher than average obesity rates, particularly in the Pacific and Caribbean. Several are also world-leaders in per capita volumes of calories purchased from SSBs, notably Saudi Arabia (145.7 kcal/capita/day), Chile (160.8 kcal), and Mexico (148 kcal).^5^ Experts recommend that taxation rates should be set at non-trivial levels (ie, 10%-20% or higher) to have meaningful impacts on consumer behavior. However, most jurisdictions are well below the mark indicating significant potential for further increases.^6-8^

**Figure 2:**
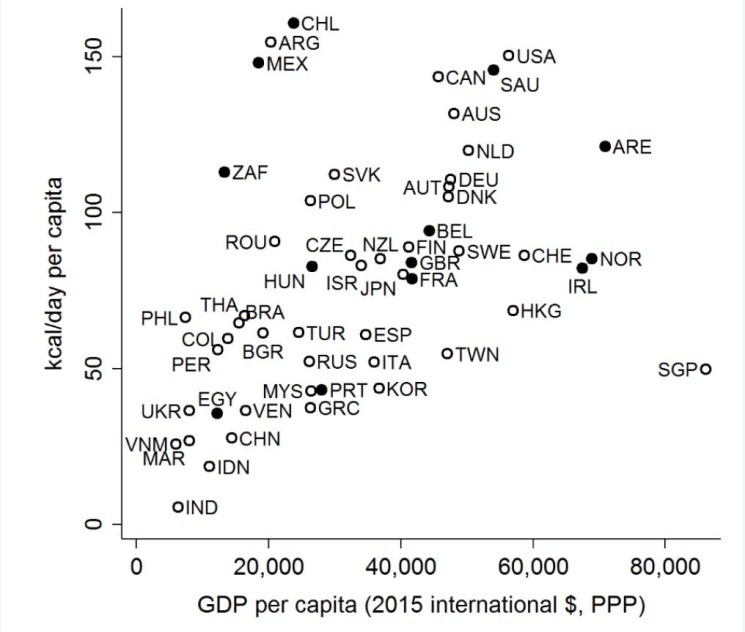
Calories Sold Per Capita Per Day From SSBs Versus GDP Per Capita in Largest Markets With an Existing Tax or Soon to Adopt a Tax (Black Dots), and Those Without (White Dots).
Note: This figure was prepared using data from the Euromonitor Passport Nutrition database^5^ and a method used in earlier studies^8,10^; GDP per capita is given as PPP and in 2015 international dollars to allow for comparability between countries.

Abbreviations: GDP, Gross domestic product; PPP, purchasing power parity; SSBs, Sugar-Sweetened Beverages.

Outside of these countries where is the untapped public health potential for SSBs greatest? One approach is to identify the countries with high levels of calories purchased from SSBs but with no tax in place. To do this we used market sales data^5^ and a method used by others in analyses of ultra-processed food and beverage markets.^8,10^ This identifies Argentina, Australia, Austria, Canada, Denmark, Germany, the Netherlands, Poland, Slovakia, and the United States (Figure 2) among others. Another approach is to consider countries with large population sizes where even small reductions in per capita SSB consumption could lead to significant population health gains. Among highly populated middle-income countries (>50 million people) only Mexico, Egypt, and South Africa have enacted SSB taxes. Brazil and several other Latin American countries, the Philippines and Thailand standout with high levels of calories purchased from SSBs but without taxes yet in place.

### Under What Conditions Are Governments More Likely to Adopt and Sustain SSB Taxes?

Recent case studies involving several of the jurisdictions with SSB taxes in place provide valuable lessons for public health advocates and policy-makers looking to follow suit. Political science theories and studies on the determinants of political priority for health issues also offer important insights.^11,12^ In this section we summarize some key findings.

*Financial reform and/or fiscal need as policy windows*: The ideological persuasion of government does not appear to influence the likelihood of tax adoption, having been almost equally as common under more left-wing (eg, social-democratic) as under more right-wing (eg, liberal-conservative) governments.^13^ What appears to be most important is a government commitment to financial reform and/or moments of fiscal need when governments are favorable to adopting new revenue streams. For example, previous taxes have been adopted within broader tax system reforms (eg, Mexico, South Africa), to address budgetary shortfalls during financial crisis (eg, Hungary), and to off-set tariff-revenue reductions following trade liberalization (eg, Fiji).^13^ Similar situations are likely to present significant windows of opportunity for tax proponents. They might find influence with powerful finance actors (eg, ministries of finance) when highlighting the revenue-raising potential of SSB taxes, including for the purpose of broadening health systems financing models.^14^ Or, as in the case of Mexico,^15^ when they are capable of sensitizing financial policy reform agendas to public health nutrition objectives.

*Framing:* Certain messages (ie, frames) appear to be effective in raising awareness and generating support for SSB taxes in different contexts. In addition to highlighting their revenue-raising potential, framing taxes as a health promotion measure may generate further political support.^16,17^ For example, in Mexico, a multi-pronged media campaign highlighting the health and economic burden of obesity and diabetes, the uniquely harmful contribution of SSBs to these problems, and the presentation of evidence-based solutions with an emphasis on tax adoption were influential with the general public and legislators.^15^ More immediate and graphic harms of SSBs on dental health in both adults and children may also be persuasive in making the case for action to policy-makers. Support also tends to increase when there is a clear commitment to earmark tax revenues for health and/or other social initiatives.^3,13,16,17^ Within the framework of the Sustainable Development Goals and United Nations Decade of Action on Nutrition there are many potential targets for hypothecation (eg, universal coverage of essential nutrition actions, food distribution and social protection programs, water and sanitation initiatives and so on).^18^ Pro-tax messages may have greater salience when they come from trusted sources (eg, well-regarded public-interest organizations, celebrities such as Jamie Oliver, or local community members),^3,13,15^ and when highlighting the predatory nature of soda industry marketing practices on children.^11,13,19^

*Civil society mobilization:* In several (but not all) cases proponents of soda taxes have established broad-based advocacy coalitions to raise public awareness, pool resources and generate stronger leverage with decision-makers. Such coalitions can draw upon a diversity of strategies and tactics. In Mexico, for example, an alliance of advocacy organizations, professional lobbyists and academic institutions initiated a multi-pronged media campaign to influence public opinion, developed evidence-based framing strategies, and lobbied government officials and newly-elected legislators. This coalition drew upon the financial and technical support of key international organizations including Bloomberg Philanthropies, the Pan American Health Organization, and the WHO. Research institutions played a key role in generating and synthesizing evidence, including the evaluation of tax effectiveness.^15^

*Anticipating and countering opposition:* As Roache and Gostin point-out SSB industries have powerfully impeded support for taxes in many jurisdictions. This includes direct lobbying of legislators, legal challenges prior to and following tax implementation, and anti-tax media campaigns emphasizing the anticipated harms to business and jobs, and anti-government (eg, nanny-state) sentiments. Arguments put forward mirror those of the tobacco industry.^20^ These strategies are to some extent predictable and may be countered. For example, another industry strategy is the framing of soda taxes as regressive (ie, as unfairly burdening low-income groups). Several evidence-based frames may be deployed by tax proponents to counter such arguments, for example: earmarking revenues for targeted health and social spending can off-set any regressive impacts of a SSB tax on low-income groups^13,16,21^; designing taxes so as to incentivize product reformulation (eg, as in the case of the UK SSB levy) can reduce the cost passed onto consumers and delivers benefits across the population equitably^16^; and the health and economic burden of SSBs on low-income consumers is even more regressive than SSB taxation.^3^

## Strengthening Capacities to Design and Implement Effective Sugar-Sweetened Beverage Taxes

Governments must have the commitment to implement SSB taxes. But they must also have the capacity to design and effectively administer them. Here we consider potential technical and legal capacity challenges and how public health actors might support governments in overcoming them.

*Technical capacities:* The design of SSB tax systems can present several technical challenges for governments. Key considerations for increasing tax effectiveness and survivability include the establishment of clear policy objectives, demarcation of taxable product categories with links to available evidence, the administrative complexity and effectiveness of different tax structures (ie, adopting specific excise taxes, ad-valorem excise taxes and value-added taxes separately or in combination), administrative capacities of implementing agencies, and the establishment of robust monitoring and evaluation systems.^13,21,22^ Roache and Gostin point out the WHO’s important role in providing technical assistance, and sharing information and country experiences. Recent technical reports provide a strong foundation here.^7,21^ Future activities might include the development of clear guidance on the implementation of price and tax measures for SSBs specifically and/or healthy diets more broadly (eg, similar to Article 6 of the Framework Convention on Tobacco Control). The World Bank can also play an important role given its close engagement with ministries of finance and its expertise in generating and disseminating evidence.^4^ Monitoring and evaluation activities by research institutions are particularly important to tax survivability because, as in the case of Mexico, evidence demonstrating tax effectiveness can be used to counter ongoing opposition.^4^

*Legal capacities:* Collaboration with lawyers during the design, implementation and evaluation of SSB tax regulations can help to ensure that policies not only achieve their health objectives, but also comply with domestic and international law. The significant funds invested by the beverage industry to defeat SSB taxes through litigation thwart or delay progressive regulation in both the jurisdiction directly affected and beyond by inciting what is sometimes called ‘regulatory chill.’ This may be particularly so for low- and middle-income countries without the legal capacity or financial resources to defend such challenges, even where the claims made lack merit. Tobacco control experience highlights tax opponent’s use of challenges to public health regulations on the basis of their failure to comply with relevant procedural or administrative requirements in domestic or international law.^20^ Understanding these requirements can assist policy-makers to comply with due process in making tax regulations, for example by allowing appropriate opportunity for consultation, or a reasonable period for implementation.

Lawyers and public health actors can also collaborate to ensure legislation objectives are framed strategically to reflect available scientific evidence, and distinctions between taxed and non-taxed products (ie, SSB definitions) contained in any regulation are defensible on public health grounds.^23^ Much can be gained from sharing regulatory-best practice, for example in the way Mexico’s legislation was crafted to ensure constitutionality.^15^ Explicit recognition of the role of SSB taxes as only one aspect of comprehensive policies to promote healthier diets may also be useful in the event of legal challenge that involves a court’s assessment of the policy’s effectiveness. Furthermore, positioning SSB taxes within regional and global policy frameworks on noncommunicable diseases may strengthen both the political and legal mandates for action, and contribute evidence of international consensus in the event of any legal challenge.^24,25^ At the same time, different legal, economic and social contexts in each jurisdiction will mean regulation will require tailoring.^25^

## Conclusion

The untapped potential for accelerating the worldwide adoption of SSB taxes is substantial. There is now a strong and growing body of evidence on *why* governments should adopt such taxes, particularly with regards to their value as a policy instrument for attenuating SSB consumption, raising revenue and driving product reformulation. Arguably more attention is needed by the public health community with regards to *how* governments might be encouraged and supported to implement SSB taxes in the future. Continued capacity-building efforts and sharing of international best-practice will generate a clearer understanding of the conditions under which governments are more likely to make SSB tax implementation a political priority, and the technical and legal capacities required to ensure such policies are effective.

## Ethical issues

Not applicable.

## Competing interests

Authors declare that they have no competing interests.

## Authors’ contributions

PB wrote the first draft of the manuscript with inputs from AJ and AMT. AJ collected data and prepared Figure 1 with assistance from Alexander Baldock. PB collected and analyzed the SSB data presented in Figure 2. All authors contributed to iterations of the manuscript and approved the final version.

## Authors’ affiliations

^1^Institute for Physical Activity and Nutrition, School of Exercise and Nutrition Sciences, Deakin University, Geelong, VIC, Australia. ^2^The George Institute for Global Health, University of New South Wales, Sydney, NSW, Australia. ^3^The Charles Perkins Centre, University of Sydney, Sydney, NSW, Australia. ^4^Menzies Centre for Health Policy, Charles Perkins Centre, University of Sydney, Sydney, NSW, Australia.
